# Pandemic (H1N1) 2009 and Hajj Pilgrims Who Received Predeparture Vaccination, Egypt

**DOI:** 10.3201/eid1707.101484

**Published:** 2011-07

**Authors:** Amr Kandeel, Michael Deming, Eman Abdel Kereem, Samir El-Refay, Salma Afifi, Mohammed Abukela, Kenneth Earhart, Nasr El-Sayed, Hatem El-Gabaly

**Affiliations:** Author affiliations: Ministry of Health, Cairo, Egypt (A. Kandeel, E. Abdul Kereem, S. El-Refay, M. Abukela, N. El-Sayed, H. El-Gabaly);; Centers for Disease Control and Prevention, Atlanta, Georgia, USA (M. Deming);; US Naval Medical Research Unit No. 3, Cairo, Egypt (S. Afifi, K. Earhart)

**Keywords:** Hajj, influenza, pandemic (H1N1) 2009, vaccine, viruses, Egypt, dispatch

## Abstract

In Egypt, vaccination against pandemic (H1N1) 2009 virus was required of pilgrims departing for the 2009 Hajj. A survey of 551 pilgrims as they returned to Egypt found 542 (98.1% [weighted]) reported receiving the vaccine; 6 (1.0% [weighted]) were infected with influenza virus A (H3N2) but none with pandemic (H1N1) 2009 virus.

The annual Hajj pilgrimage brings >2.5 million pilgrims from >160 countries to Mecca and Medina in Saudi Arabia during a 1-week period (*1*). In the past, 6.0%–9.8% of Hajj pilgrims with acute respiratory tract infection have been found to have influenza (*2**–**4*). Seasonal influenza vaccine is recommended for pilgrims by Saudi Arabia’s Ministry of Health (*5*), but vaccination coverage has been low (*4**,**6*).

## The Study

The Hajj occurs during the twelfth month of the Islamic calendar, a lunar calendar that shifts 11 days earlier each year. The 2009 Hajj took place during November 25–28, (1430 AH), when the Northern Hemisphere was experiencing high pandemic (H1N1) 2009 virus activity, raising concern that the gathering could further contribute to the global spread of pandemic (H1N1) 2009. Health experts meeting in Jeddah in June 2009 made recommendations for reducing the pandemic’s effects during the Hajj (*7*). The value of predeparture vaccination was recognized, but it was thought unlikely that pandemic vaccine would be available in sufficient quantities to have a major effect on transmission during the Hajj. Egypt received the vaccine in time to begin vaccinating pilgrims on November 3, 2009, and required predeparture vaccination for all pilgrims to protect them against illness and reduce the importation of pandemic (H1N1) 2009 into Egypt when they returned. Egypt also enforced the Jeddah group’s recommendation that the 2009 Hajj pilgrimage be made only by persons 12–65 years of age.

Pandemic (H1N1) 2009 virus was first detected in Egypt in June 2009. It did not become the predominant influenza virus causing influenza-like illness (ILI) in Egypt’s ILI sentinel surveillance system until mid-November 2009 (*8*).

Approximately 80,000 Egyptians make the Hajj pilgrimage each year, usually returning to Egypt a few days to a few weeks after it. In all, 70%–80% of pilgrims arrive at Cairo International Airport or Port Tawfiq, near Suez (Ministry of Health, Egypt, unpub. data). During the peak return period, 7 or 8 flights arrive each day from Jeddah, with an average of 200–250 pilgrims per flight, and Port Tawfiq receives 1 ship per day from Jeddah with ≈1,000–1,200 pilgrims. The goal of the survey was to measure the prevalence of pandemic (H1N1) 2009 virus infection among returning pilgrims.

The survey was conducted by a team of 2 epidemiologists, 2 health workers, and 2 laboratory technicians from the Preventive Sector, Egyptian Ministry of Health, during the peak return period. Every tenth pilgrim on the ship from Jeddah arriving at Port Tawfiq on December 14, 2009, was selected for the survey sample without regard to illness status. At Cairo International Airport, pilgrims were selected from among all pilgrims on the 9 flights from Jeddah arriving during 9 AM–9 PM on December 10–12, 2009. Because the survey was conducted at the baggage-claim area, probability sampling proved to be difficult. With instructions to choose pilgrims throughout the area around the carousel without regard to age, sex, or illness status, the team selected a convenience sample of ≈50 pilgrims from each flight.

After providing verbal consent, pilgrims were asked their age, in which governorate they lived, and whether they had been vaccinated against pandemic (H1N1) 2009 virus. Their oropharynx was then swabbed, and swab specimens were placed in viral transport medium and kept in liquid nitrogen until transfer to the Ministry of Health’s Central Public Health Laboratory. All specimens were tested at the US Naval Medical Research Unit No. 3 in Cairo by real-time reverse transcription PCR (rtRT-PCR) for influenza A viruses, and all specimens positive for influenza A viruses were tested for influenza A subtypes, including pandemic (H1N1) 2009 virus, by rtRT-PCR, according to guidelines of the World Health Organization and the Centers for Disease Control and Prevention (Atlanta, Georgia, USA) (*9*). Testing was not performed for influenza B. A recent study found oropharyngeal swab samples to be more sensitive than nasopharyngeal swab samples for detecting pandemic (H1N1) 2009 virus by rtRT-PCR (*10*).

Results were weighted according to probabilities of selection within the ship and each plane, which were considered separate strata. Data were analyzed with PROC SURVEYFREQ in SAS version 9.1 (SAS Institute Inc., Cary, NC, USA).

In all, 559 pilgrims were selected for the survey sample. Seven pilgrims refused to participate, and interview data were missing for 1 pilgrim, leaving 551 pilgrims in the analysis: 206 pilgrims from 4 flights on December 10, 219 pilgrims from 5 flights on December 12, and 126 pilgrims from the ship arriving December 14. The stated age of 549 (99.6%) of pilgrims in the sample was in the allowed age range of 12–65 years. Most were from the Cairo metropolitan area (43.8%) or Lower Egypt (51.0%); these areas were overrepresented compared with the proportion of the national population living in them (Table). All but 9 (98.1%) pilgrims reported receiving a predeparture vaccination against pandemic (H1N1) 2009 virus. No association was found between predeparture vaccination status and sex (p = 0.38), age (<55 years vs. >55 years) (p = 0.95), or area of residence (Cairo metropolitan area vs. outside this area) (p = 0.20). In all, 6 (1.0%, 95% confidence interval 0.2%–1.7%) pilgrims tested positive for influenza A. All had subtype H3N2. No pilgrim had positive results for pandemic (H1N1) 2009 virus.

**Table:** Demographic characteristics, vaccination history, and influenza A infection prevalence among 551 pilgrims returning from the Hajj, Egypt, 2009

Characteristic	No. pilgrims (weighted %)
Age group, y	
<30	11 (2.2)
30–39	66 (12.4)
40–49	141 (23.9)
50–59	219 (40.2)
60–69	114 (21.2)
>70	0
<12*	1 (0.3)
>65*	1 (0.1)
Gender	
M	311 (57.8)
F	240 (42.2)
Area of residence†	
Coastal Egypt	12 (3.1)
Lower Egypt	298 (51.0)
Cairo metropolitan area	232 (43.8)
Upper Egypt	9 (2.1)
Verbal history of pandemic (H1N1) 2009 vaccination	
Yes	542 (98.1)
No	9 (1.9)
Pilgrims infected with influenza A virus, by subtype‡	
Seasonal (H1N1)	0
Seasonal (H3N2)	6 (1.0)
Pandemic (H1N1) 2009	0

*Outside the age limits recommended for Hajj pilgrims by the Ministry of Health, Saudi Arabia, and enforced by Egypt’s Ministry of Health. †The proportion of the national population living in these 4 areas according to the 2006 census was 11.3%, 34.3%, 19.4%, and 34.9%, respectively (*11*). The areas were formed by grouping Egypt’s governorates as follows: Coastal Egypt: Alexandria, Damietta, Ismailia, Matrouh, North Sinai, Port-Said, Suez, Red Sea; Lower Egypt: Behera, Dakahlia, Gharbia, Kafr-ElSheikh, Menoufia, Sharkia; Cairo metropolitan area: Cairo, Giza, Kalyoubia; Upper Egypt: Aswan, Asyout, Beni-Suef, ElWadi ElGidid, Fayoum, Helwan, Luxor City, Menia, Qena, South Sinai, Suhag, and 6th October. ‡By real-time reverse transcription PCR testing.

This finding supports the conclusion that returning pilgrims likely contributed little to ongoing pandemic (H1N1) 2009 transmission in Egypt and is consistent with the intended effects of the predeparture vaccination requirement. At the time of the 2009 Hajj, pandemic (H1N1) 2009 was overwhelmingly the most common influenza virus in the Northern and Southern Hemispheres (*12*), and to our knowledge, few countries required predeparture vaccination against it. Thus, we expect that pilgrims were exposed to this virus during the Hajj, but the extent of exposure is uncertain. The only data of which we are aware that have been released thus far on the extent of such exposure were provided by the Minister of Health, Saudi Arabia, who announced on the last day of the Hajj that pandemic (H1N1) 2009 had been diagnosed in only 73 persons during the Hajj (5 of whom died) (*13*). Haworth et al. have argued that a much larger number of cases is likely, on the basis of the expected case-fatality ratio among the Hajj pilgrims and on modeling results (*14*).

Our survey had several limitations. First, the results may not apply to pilgrims who returned to Egypt before or after the survey period. Second, pilgrims from Lower Egypt and coastal Egypt were underrepresented in the survey sample. Third, convenience sampling was used to select pilgrims arriving by plane. Fourth, unvaccinated pilgrims may have been reluctant to tell interviewers they had not been vaccinated because predeparture vaccination was required. Finally, some pilgrims may have been infected with pandemic (H1N1) 2009 virus shortly before swab samples were obtained but were not yet shedding virus.

## Conclusions

Egypt demonstrated that it could implement a predeparture vaccination requirement despite late arrival of the vaccine, and our survey found no evidence that pilgrims returned to Egypt with pandemic (H1N1) 2009 virus infection during the peak return period. These results may prompt other countries to consider a similar influenza vaccination policy before the Hajj and other mass gatherings where amplification of influenza virus transmission is a major threat. Studies of vaccine effectiveness, cost-effectiveness, and cost-benefit in these settings would provide additional useful information.
